# Toward a Membrane-Centric Biology

**DOI:** 10.3389/fimmu.2020.01909

**Published:** 2020-09-09

**Authors:** Yan Shi, Hefei Ruan

**Affiliations:** ^1^Tsinghua-Peking University Joint Center for Life Sciences, Tsinghua University, Beijing, China; ^2^Department of Basic Medical Sciences, Tsinghua University, Beijing, China; ^3^Institute for Immunology, Tsinghua University, Beijing, China; ^4^Beijing Key Lab for Immunological Research on Chronic Diseases, School of Medicine, Tsinghua University, Beijing, China; ^5^Department of Microbiology, Immunology and Infectious Diseases, Snyder Institute, University of Calgary, Calgary, AB, Canada

**Keywords:** plasma membrane, lipid rafts, evolution, receptor ligand model, lipid interaction, receptor activation mechanism

## Abstract

With advancements of modern biophysical tools and superresolution imaging, cell biology is entering a new phase of research with technological power fitting for membrane dynamics analyses. However, our current knowledge base of cellular signaling events is mostly built on a network of protein interactions, which is incompatible with the essential roles of membrane activities in those events. The lack of a theoretical platform is rendering biophysical analyses of membrane biology supplementary to the protein-centric paradigm. We hypothesize a framework of signaling events mediated by lipid dynamics and argue that this is the evolutionarily obligatory developmental path of cellular complexity buildup. In this framework, receptors are the late comers, integrating into the pre-existing membrane based signaling events using their lipid interface as the point of entry. We further suggest that the reason for cell surface receptors to remain silent at the resting state is via the suppression effects of their surrounding lipids. The avoidance of such a suppression, via ligand binding or lipid domain disruption, enables the receptors to autonomously integrate themselves into the preexisting networks of signaling cascades.

## Introduction

The main goal of this piece is to gather sufficient consensus regarding how biophysicists, or other specialists so inclined, may approach life science research with a stronger footing of legitimacy. In recent years, with advancements in superresolution imaging and computational biology, biophysicists are given enormous probing power in our life science work. In comparison, traditional biologists using more conventional tools are still making ground-breaking discoveries at a pace appreciably faster than most of us. A sobering dichotomy is evident that findings made with biophysical approaches are being regarded as “supplemental” to other paradigms. Using T cell biology as an example, whereas many papers have been published in this area regarding membrane behavior upon T cell receptor activation, the whole theoretical framework of T cell activation can be completely explained without any reference to biophysical properties. Points of interest in biophysics or membrane biology are generated to explain the details of how polypeptides work. While we know for certain that this cannot be true, our strongest protest may be the insistence that “no signaling can be fully understood without its membrane platform.” *C'est la vie*, such a defensive stand will not change the “outsider looking-in” mentality. We need to move biophysics and membrane biology to the frontlines of biological research. The questions are “why hasn't it happened?” and “what is the main roadblock?”

## Main Text

The current state of biological research is protein-centric. The cause lies in its history and the availability of investigative tools. Yet, conceptual inertia is not beyond reproach. Our inability to delineate biological events with models built from membrane biology is the core deficiency. It is time that we collectively reflect on this dilemma. In this opinion piece, we make a call for change.

### Membrane Structure, Critical Behavior, and Their Preservation in Biology

Let's start with the eukaryotic membrane. In the 70s, Singer and Nicolson presented the mosaic model in which the membrane bilayer was regarded as a fluid mixture of lipids and proteins (1). With many years of work into the heterogeneity of vesicular and plasma membranes, Kai Simons et al. in 1997 proposed the concept of ordered and disordered membrane phases generically known as the lipid raft theory (2). Aki Kusumi, mainly using particle tracking, refined this model with an additional detail that lipid domains are stabilized by membrane lipid binding to cortical cytoskeleton, or the picket and fence model (3). As those theories are discussed at length elsewhere and readers of this writing are well versed in this stream of concepts, we simplify our discussion with the most accepted membrane model (4). Eukaryotic membrane inner leaflets are occupied by mostly phospholipids with negative charge and are active in signal exchange in abundance. In comparison the outer leaflets are structurally dynamic. There, sphingolipids and gangliosides are more enriched, perhaps due to their enlarged head groups more suited to the positive curvature. Cholesterol, nimble in size and low in charge, is free to move laterally (5) or change leaflets via overcoming the energy barrier set by hydrophobic core of the bilayer (6). The rendezvous of sphingolipids, cholesterol, and saturated phospholipids at physiological temperatures forms the structure of ordered lipid domains. The formation of ordered vs. disordered lipid domains can be explained by the combined entropic diffusion and energy conservation in special lipid pairing (7, 8). Remarkably, this feature is common to all eukaryotes despite the vast different collections of lipid species in distinct cell types. About 5% of genes are dedicated to maintain it (4). As current efforts have not been able to fully mimic domains found in live cells with defined lipids, the remarkable preservation implies an extreme cellular dedication in their maintenance. This point alone should give us a strong clue that this is something central to all aspects of eukaryotic biology.

Another intrigue of the lipid domains is the critical behavior which refers to the state where at physiological temperature, lipid domain formation (demixing) and dissolution (mixing) are at a critical point (7). This feature, coupled to cytoskeletal association, was vividly demonstrated with STED superresolution microscopy, and with a clear linear correlation to the temperature (9). Such a delicate feature allows large phase transition with minimal energy input. For instance, minute disturbance of receptor ligand interaction may force such a phase change, an ingenious system of signal amplification (receptor ligation can be viewed as a localized suppression of entropy, or cooling). We shall return to this point later. Nevertheless, it should be noted that such a behavior is unimaginable in a cohort of protein molecules.

### Current Status of Understanding

Since the proposal of lipid rafts, biologists have tried to incorporate this feature into their models of membrane signaling. To circumvent the optical diffraction limit which makes visual observations of resting cell lipid rafts impossible, two surrogates have been developed. One is to isolate detergent-resistant membrane domains, hoping to capture proteins associated with or free from lipid rafts at the moment of cell lysis (10). The other, used by some, is to observe domain coalescence at the point of “signalosome” formation (11) or visible lipid domains found on GPMV (12). One of the most influential conclusions is the partition of protein molecules into different phases of membrane domains. From those experiments, it was understood that the transition of those protein molecules with reference to lipid domains is associated with their state of activation. Some are activated in disordered phase, such as EGFR (13), while in others transition into or residence inside the ordered domain is required for their activation, such as death receptor Fas, IFNγR, and Wnt receptor (14–16). In addition, protein signaling complex formation with the participation of numerous components is also controlled by the coalescence of lipid rafts, such as in TCR activation (17). Regardless of the study subject, in a protein-centric world, those events are regarded as the consequence of receptor ligation and protein–protein interaction, which is taken as the driving force of lipid domain alteration.

Those observations, however, are not without their own peril. First, the selection of detergents has a tremendous effect on the observed association, demanding caution in data interpretation. Perhaps more importantly, this “snap photo” approach will leave out spatial temporal regulation. Using TCR as an example, the signaling is mediated by TCR ligation by the MHC/peptide complex, yet the signal is initiated at Src family kinase activation of the tyrosine residues in the ITAM motifs. Thereafter, the signal has several bifurcations or multiplications; some of downstream events such as Lck and LAT are clearly dependent on lipid domains (18), while others such as TCR itself and CD45 are not (19, 20). Likely due to those technical limitations, some conclusions are not always in agreement. In a remarkable demonstration of collegiality among biophysicists, those differences are accepted as limits of one's own research unable to explain seemingly contradicting results. In fact, those differences *en masse* reflect the lack of more sophisticated tools as well as a biophysical explanation of how membranes work in this setting. Happily, the constraint posed by tool selection is being rapidly lifted in recent years. A particular case in point is the newly gained ability to study the dynamics of lipid rafts on the cell membrane, which in our opinion is the technological foundation to introduce membrane dynamics into core concepts of biology.

### Evidence of Membrane Lipid Interface Is a Biological Switch

Imagine a simplest eukaryotic cell with no cell surface receptor and driven by a few signaling pathways that support the basic biology. All those regulations are anchored to membrane sensing. Earliest multicellular animals were found about 600 million years ago. Shortly after, about 550 million years ago, life rapidly diversified during the Cambrian explosion. Around that time two rounds of whole genome duplication likely provided a genetic playground for the emergence of vertebrates (20). It is hard to imagine that receptor ligand interactions would have been the dominant way of communication prior to these junctures. The definition of receptor-ligand interaction is that they must be evolutionarily coupled. As a single cell is exposed to an unmalleable environment, this co-evolution lacked a driving force. On the flip side, receptor-independent sensing of environment, such as phagocytosis (21, 22), was a daily occurrence that had propelled the evolution for at least 1.4 billion years. Many prominent signaling pathways came before this time, including GTPase (23), MAPK (24), phagocytosis (21), TNFRF (25), Jak/Stat (26) pathways, metalloproteases, (27) and metabolic events with a possible exception of Wnt pathway (28). All those pathways are regulated by membrane events. In our own research, we first discovered that solid particle binding to plasma membrane induces the accumulation of lipid rafts which triggers phagocytosis (29, 30). Based on this finding, we further revealed that immune receptors had evolved out of a primordial phagocytic signaling that uses the membrane anchoring protein moesin to sense the PIP2 accumulation in the inner leaflet as a result of particle binding. Moesin binding to PIP2 opens its ITAM motif, for downstream signaling, including Syk and PI3K. Remarkably, all those events are used verbatim in immune signaling of all classes, including BCR, TCR, and Fc receptors (21). Therefore, the adaptive immunity hijacked the machinery of the ancient phagocytosis following membrane sensing. If the intracellular events are regulated by membrane activities, what argues against the notion that modern immune receptors initiate signaling with the same mechanism?

**Figure 1:**
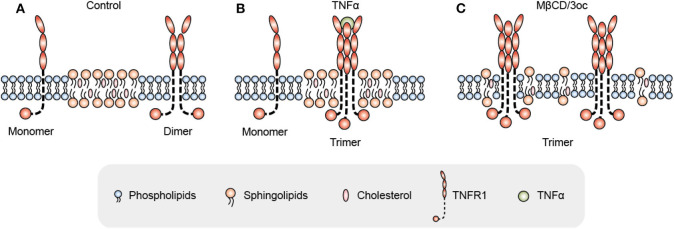
Proposed mechanism for TNFR1 signal activation initiated as a negotiation between the receptor and its surrounding lipid species. **(A)** In the resting state, TNFR1 mainly exists as monomers and dimers on the cell membrane under the suppression of dense lipid rafts. **(B)** Under TNFα ligand stimulation, TNFR1 negotiates with lipid domains by changing its transmembrane domain conformation, and then enters lipid rafts for trimerization. **(C)** When the lipid rafts are disrupted by MβCD or 3oc [N-(3-oxododecanoyl) homoserine lactone], TNFR1 spontaneously trimerizes and activates signals without ligands as the membrane suppression is relieved.

**Figure 2:**
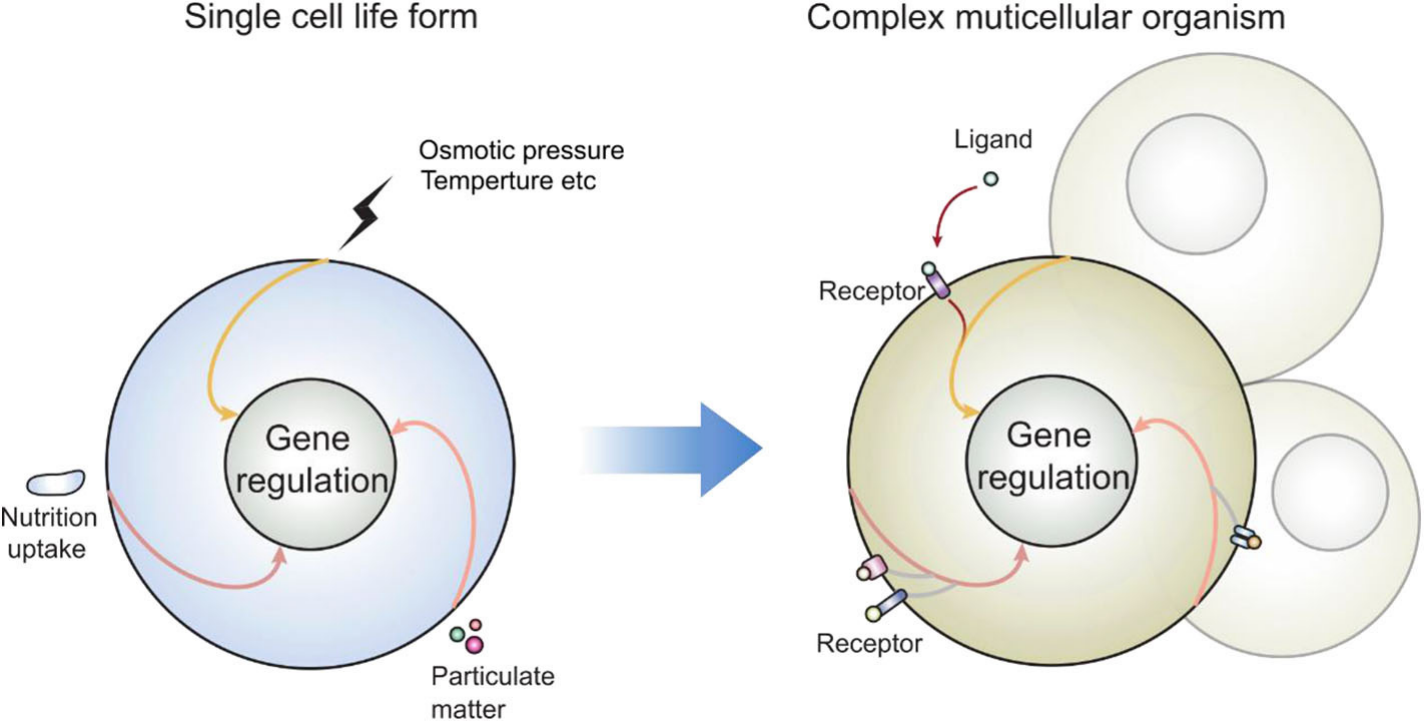
How receptors may enter the preexisting signal cascades: as many signaling events present in modern day eukaryotic cells predate the multicellular life forms, those enclosed single cells interacted with the environment via lipid membrane. Those signal cascades are therefore coupled to membrane sensing, particularly in response to lipid domain alteration. New receptors cannot reinvent a new signal cascade; rather than producing protein molecules as adaptors, they can also regulate their own lipid interface, which is the legitimate and built-in mechanism of cell activation.

Let's look at another set of events observed by most if not all whom have attempted: the consequence of lipid domain perturbation, mostly in the form of cholesterol depletion. EGFR (31–33), TNFR1 (34), TLRs (35, 36), TCR (19, 37, 38), TGFβR (39–41), and shedding events (42–44) are triggered by lipid domain disruption. Figure 1 illustrates our own findings. There are certainly good examples where clustering toward lipid rafts is induced by ligand binding. Linked to the observation that those receptors are sequestered in their own lipid environment at the resting state, one can reasonably predict: 1. Some receptors are self-activating depending on the lipid environment or phase transition. 2. For one receptor to be accepted as useful contributor to biology, it would have to obey the suppression of the membrane. Therefore, membrane lipid phases are an ingrained tool of suppression of receptors. Kai Simons noted that due to the size of lipid domains, each raft would contain very few polypeptides (45). Therefore, those receptors are blocked by their spatial separation. Once the blockage is released, such as in the case of domain disruption, they become activated. Fessler and Parks used a number of examples to show how lipid perturbation itself is sufficient to activate many receptors (46). Yet, they stopped at the last step to connect the final dots that lipid perturbation-mediated receptor activation and ligand/receptor-based activation may be fundamentally the same at least for some receptor families. To extrapolate this idea further, we can imagine that a receptor ligation interaction, rather than bringing in certain conformations to accommodate their interaction binding partners, could easily regulate its lipid interface to avoid the suppression. In light of the critical behavior in membrane lipids, such a minute change can cause the clustering of signaling molecules as a consequence of lipid domain alteration at the energy level fitting for receptor ligand interaction. This hypothesis, with modern tools, should be testable *in situ* or on a model membrane without the need of biological feedback, which often causes protein-centric analyses becoming embroiled in incessant cycles of amplification. If a receptor/lipid interface event is established as being autonomous, this binary regulation certainly carries an enormous power of prediction, with a simplistic elegance not seen in sometimes chaotic search of how each protein receptor works with its downstream partners (Figure 2). At minimum, it explains why several thousand signaling receptors can stay silently together on a cell, and during an activation event, several signaling cascades are triggered at the same time, as those involved are likely gated by their own membrane sequestration. This is not to say that receptors are mere puppets in this chain of events; they certainly play their role in the clustering and their own complex formation as they also possess ranges of lipid specificities and membrane dynamics (47, 48). But the activation initiation point can be explained by lipid receptor interface, or the sum of “collectives” of protein-lipid interaction following the introduction of a ligand.

### Toward a Simple Beginning

Our lab has preliminary data to suggest that “suppression avoidance” is a core mechanism of some cell death receptors, and the cellular signaling is initiated at the simple phase change between the receptor and its surrounding lipid species. This type of effort, while fulfilling the common wisdom that receptor activation is responding to its ligand, is probably also suited to explain some activation triggers, particularly those that are not protein in nature. In such a scenario, many “ligands” can activate their “receptors” via lipid alteration without the need of direct engagement. For instance, a long acyl chain fatty acid can alter the domain features, which allows a particularly strong signaling receptor to become activated in response to lipid domain change. Macroscopically, this would look like a perfect receptor/ligand interaction. Current dogma would require the search of how this pair of receptor/ligand works, but the “suppression avoidance” model would relieve ourselves from this futility. In fact, some of the low-hanging fruits should be easy to spot.

We are not arguing against the vast network of protein signaling in biology. However, from an evolutionary perspective, membrane triggered events should be highly relevant and they set the basic signaling principles in the cell. The numerical imbalance between the vast number of cell/vesicle surface receptors and limited signaling pathways clearly tells us that the former hijacked the latter, to develop the mesmerizingly complex activation patterns in modern eukaryotic cells. Similar to the preservation of amino acid codons, those basic signaling events cannot be altered in the biological continuum. Then it is reasonable to question how the late comers, the receptor-ligand interaction, came into the theme. For our purpose, if they also use the lipid interface as the initiation point, then we have the theoretical prowess to establish a model toward a membrane-based biology, to smooth out rough edges and peculiarities in the protein-centric paradigm.

This opinion piece may be deemed inaccurate or even false in the future. However, as a research discipline with cutting edge tools and deals with some of the most autonomous events that formed the platform for other late developed biology, our collective attempt to create a landscape for this new frontier, no matter how juvenile at the beginning, is certainly worthwhile.

## Data Availability Statement

The original contributions presented in the study are included in the article/supplementary material, further inquiries can be directed to the corresponding author/s.

## Author Contributions

YS conceptualized the theory and wrote the manuscript with assistance from HR. All authors contributed to the article and approved the submitted version.

## Conflict of Interest

The authors declare that the research was conducted in the absence of any commercial or financial relationships that could be construed as a potential conflict of interest.
